# Pulmonary Safety Profile of Esc Peptides and Esc-Peptide-Loaded Poly(lactide-co-glycolide) Nanoparticles: A Promising Therapeutic Approach for Local Treatment of Lung Infectious Diseases

**DOI:** 10.3390/pharmaceutics14112297

**Published:** 2022-10-26

**Authors:** Floriana Cappiello, Bruno Casciaro, Maria Rosa Loffredo, Elena Puglisi, Qiao Lin, Dandan Yang, Gemma Conte, Ivana d’Angelo, Francesca Ungaro, Loretta Ferrera, Raffaella Barbieri, Laura Cresti, Alessandro Pini, Yuanpu Peter Di, Maria Luisa Mangoni

**Affiliations:** 1 Department of Biochemical Sciences, Laboratory Affiliated to Istituto Pasteur Italia-Fondazione Cenci Bolognetti, Sapienza University of Rome, 00185 Rome, Italy; 2 Department of Environmental and Occupational Health, University of Pittsburgh, Pittsburgh, PA 15261, USA; 3 Department of Pharmacy, University of Napoli Federico II, 80131 Napoli, Italy; 4 Department of Environmental, Biological and Pharmaceutical Sciences and Technologies (DiSTABiF), University of Campania Luigi Vanvitelli, 81100 Caserta, Italy; 5 U.O.C. Genetica Medica, IRCCS, Istituto Giannina Gaslini, 16147 Genoa, Italy; 6 Biophysic Institute, Consiglio Nazionale delle Ricerche (CNR), 16149 Genoa, Italy; 7 Department of Medical Biotechnologies, University of Siena, 53100 Siena, Italy

**Keywords:** antimicrobial peptides, cystic fibrosis, lung infection, biodegradable nanocarrier, therapeutic index, transepithelial resistance, lung gene expression, mouse bronchoalveolar lavage

## Abstract

In recent years, we have discovered Esc(1-21) and its diastereomer (Esc peptides) as valuable candidates for the treatment of Pseudomonas lung infection, especially in patients with cystic fibrosis (CF). Furthermore, engineered poly(lactide-co-glycolide) (PLGA) nanoparticles (NPs) were revealed to be a promising pulmonary delivery system of antimicrobial peptides. However, the “ad hoc” development of novel therapeutics requires consideration of their stability, tolerability, and safety. Hence, by means of electrophysiology experiments and preclinical studies on healthy mice, we demonstrated that neither Esc peptides or Esc-peptide-loaded PLGA NPs significantly affect the integrity of the lung epithelium, nor change the global gene expression profile of lungs of treated animals compared to those of vehicle-treated animals. Noteworthy, the Esc diastereomer endowed with the highest antimicrobial activity did not provoke any pulmonary pro-inflammatory response, even at a concentration 15-fold higher than the efficacy dosage 24 h after administration in the free or encapsulated form. The therapeutic index was ≥70, and the peptide was found to remain available in the bronchoalveolar lavage of mice, after two days of incubation. Overall, these studies should open an avenue for a new up-and-coming pharmacological approach, likely based on inhalable peptide-loaded NPs, to address CF lung disease.

## 1. Introduction

The rising appearance of drug-resistant microorganisms has undermined the success achieved in the last century in the field of medicine via the utilization of antibiotics [1,2,3,4]. Antimicrobial peptides (AMPs) with a rapid-membrane-perturbing activity make bacteria less prone to develop resistance and constitute a promising source of new effective therapeutics [5,6,7]. Because of their biocompatibility, seldom accumulation in body tissues, and the harmlessness of their degradation products, peptides have become a unique class of therapeutic agents [8]; to date, more than 80 peptides have reached the global market [9]. In parallel, extensive research has been carried out in terms of the discovery, production, and optimization of AMPs, and an average of 70 AMPs are in preclinical/clinical evaluation [10,11]. AMPs are generally produced by all living species as key effectors of the innate immune system [12]; amphibian skin represents an invaluable wealthy storehouse of such molecules [13]. Studies conducted in our laboratory have led to the discovery of a frog-skin-derived membrane-active AMP, i.e., Esc(1-21), with bactericidal activity against both the planktonic and biofilm forms of the human pathogen *Pseudomonas aeruginosa* [14]. Nowadays, lung colonization by the sessile form of this bacterium holds a challenging threat, mostly in patients affected by cystic fibrosis (CF) [15,16], a genetic disease caused by mutations in the gene encoding the CF transmembrane conductance regulator (CFTR) protein, which controls chloride and bicarbonate transport mainly at the apical membrane of secretory epithelia, including those at the airways [17,18]. The most common mutation is the deletion of phenylalanine 508 (F508-del CFTR), which causes the production of an incorrectly folded protein that is rapidly degraded [19]. Furthermore, the small fraction of F508del-CFTR that reaches the plasma membrane [20] exhibits a defect in channel gating. As a result, the outflow of anions is inhibited, leading to increased water absorption by epithelial cells and the formation of a sticky airway mucus where inhaled microbes accumulate, giving rise to the onset of chronic pulmonary infection with serious respiratory dysfunctions.

During the last several years, by changing the stereochemical configuration of only two L-amino acids of Esc(1-21), i.e., Leu^14^ and Ser^17^, to the corresponding D-enantiomers, we discovered that the resulting diastereomer Esc(1-21)-1c is: (i) more resistant to enzymatic degradation [21] and (ii) more efficient in restoring bronchial epithelium integrity [22,23]. This last feature is not shown by any traditional antibiotic and is expected to accelerate the healing of a damaged lung epithelium, especially in CF lungs, where wound-healing processes are highly compromised [24]. Interestingly, we also discovered an unprecedented property of AMPs, which is the ability of both Esc(1-21) and Esc(1-21)-1c (Esc peptides) to act as potentiators of the CFTR with ion conductance defects [25].

However, the “ad hoc” development of novel therapeutics based on peptide antibiotics and their translation from basic research to the clinic requires consideration of several underexplored aspects associated with the effective application of AMPs. These include studies on: (i) peptides’ stability under conditions that reflect the physiology of the target site (e.g., the lung); (ii) tolerability for the assessment of a therapeutic window; (iii) identification and validation of an efficient delivery system of AMPs without eliciting undesirable local and/or systemic side effects. Previous studies highlighted Esc(1-21)-1c as the most efficient Esc peptide in reducing the lung bacterial burden in a mouse model of acute Pseudomonas lung infection, upon a single intratracheal (i.t.) instillation at a very low dosage (0.1 mg/kg, corresponding to 20 μM) with comparable efficacy to colistin, the last-resort antibiotic for the treatment of infections [26]. Nevertheless, studies aimed at evaluating Esc peptides’ local toxicity or tolerability at higher therapeutic dosages are missing. Recently, we also demonstrated how polyvinyl-alcohol (PVA)-engineered poly(lactide-co-glycolide) (PLGA) nanoparticles (PVA-PLGA NPs) represent an enticing nanoformulation for pulmonary delivery of AMPs, able to (i) assist AMP diffusion through biological barriers, such as the mucus (which becomes a thick layer in CF) and (ii) prolong AMP antibacterial efficacy against Pseudomonas-induced lung infection [27]. Conceiving PVA-PLGA NPs as a promising inhalable formulation for the treatment of lung infections, and investigating their pulmonary safety, in terms of gene expression and tissue integrity compared to the free peptides, would be highly recommended [28].

Hence, in line with the above, we initially performed studies aimed at identifying the effect(s) of peptide-based PLGA formulation in comparison to the soluble free form of Esc peptides on both (i) the integrity of CFTR-expressing epithelium and (ii) the pulmonary host response after i.t. administration in healthy mice. Afterward, we assessed the maximum tolerated dosage and stability in the bronchoalveolar lavage for the Esc isoform endowed with the highest in vivo antimicrobial effectiveness, Esc(1-21)-1c.

## 2. Materials and Methods

### 2.1. Peptides

Synthetic Esc(1-21) (GIFSKLAGKKIKNLLISGLKG-NH_2_) and its diastereomer, Esc(1-21)-1c, GIFSKLAGKKIKN(d-Leu)LI(d-Ser)GLKG-NH_2_, were purchased from Biomatik (Wilmington, NC, USA). Briefly, the peptides were assembled by stepwise solid-phase synthesis using a standard F-moc protocol and purified via reverse-phase high-performance liquid chromatography (RP-HPLC) to a purity of 95%. The molecular mass was verified by mass spectrometry (Figure S1).

### 2.2. PVA-PLGA Nanoparticle Production and Characterization

NPs containing either synthetic Esc(1-21) or its diastereomer, Esc(1-21)-1c, at a theoretical loading of 2% (2 mg of peptide *per* 100 mg of NPs) were prepared by emulsion/solvent diffusion, as previously reported [27]. Briefly, 100 μL of an aqueous solution of each Esc peptide was added to a solution of uncapped PLGA 50:50 (Resomer^®^ RG 502H, Mw 7000–17,000 Da, inherent viscosity 0.16–0.24 dL/g, Evonik, Germany) in methylene chloride (1 mL, 10 mg/mL) under vortex mixing (Reax top, Heidolph, Germany). The resulting water-in-oil emulsion was poured into 12.5 mL of ethanol to induce the production of NPs. Then, the NP dispersion was diluted with 12.5 mL of aqueous 0.1% (*w/v*) PVA (Mowiol^®^ 40–88, average Mw ∼205 000 Da, 87–89% hydrolyzed, Merck, Italy) and kept under magnetic stirring for 10 min at room temperature. The residual organic solvent was evaporated under vacuum at 30 °C (Rotavapor^®^, Heidolph VV 2000, Germany). NP colloidal dispersion was collected, adjusted to a final volume of 5 mL, and centrifuged at 7000× *g* for 20 min at 4 °C (Hettich Zentrifugen, Universal 16R, Hettich, Germany) to isolate NPs. The resulting NP pellet was diluted in ultrapure water up to the desired final concentration. When needed, Esc-peptide-loaded NPs were freeze-dried, adding trehalose as a cryoprotectant, with a NP/trehalose ratio of 1:25 *w*/*w*, as previously reported [27]. Control bare PVA-PLGA NPs were prepared in the absence of Esc peptides. 

### 2.3. Cells

The following cell cultures were employed: CFBE41o- and Fischer rat thyroid (FRT) cells with stable expression of F508del-CFTR (F508del-CBFE41o- and F508del-FRT, respectively) as well as CFBE41o- cells expressing a wild-type CFTR (wt-CFBE41o-) [29]. CFBE41o- cells were cultured in minimal essential medium (MEM), and FRT cells were cultured in Coon’s modified Ham’s F-12 medium (Sigma-Aldrich, St. Luis, MO, USA), both supplemented with 10% fetal calf serum, 2 mM L-glutamine, and antibiotics (0.1 mg/mL of penicillin and streptomycin), at 37 °C and 5% CO_2_ in 75 cm^2^ flasks. Stable expression is maintained by adding puromycin (0.5 μg/mL or 2 μg/mL for wt-CBFE41o- or F508del- CBFE41o-, respectively) and 0.6 mg/mL zeocin for FRT cell line to the complete culture medium. CF and non-CF primary bronchial cells were from the University of Pittsburgh CF center cell culture core facility. They were cultured in flasks and maintained at air–liquid interface (ALI) until being fully differentiated as polarized cells for experiments.

### 2.4. Transepithelial Electrical Resistance (TEER) Experiments 

Epithelial integrity was evaluated by measuring the transepithelial electrical resistance in CBFE41o- and FRT cells using the 24-transwell plates (24 Millicell plates PSHT010R1) and an electronic resistance system (Millicell ERS-2, EMD Millipore, Burlington, MA, USA). The medium was replaced every 3 days, and cells were used for TEER measurements after 7 days. Two days before the experiment, epithelia were incubated in their standard culture medium supplemented with 1 μM Vx-809 in the case of F508del- CBFE41o- and F508del-FRT. After 24 h, the medium was discarded, and epithelia were treated with Esc peptides, Esc-peptide-loaded PVA-PLGA NPs, or control bare PVA-PLGA NPs (in both the apical and basolateral compartments at different concentrations). After 24 h, the medium was replaced with a saline solution containing (in mM) 130 NaCl, 2.7 KCl, 1.5 KH_2_PO_4_, 1 CaCl_2_, 0.5 MgCl_2_, 10 glucose, and 10 Na-Hepes (pH, 7.4), which was added to both apical and basolateral compartments of the permeable supports. A sterile electrode was applied onto the apical side of the transwell insert containing the cells with/without treatments, and TEER was measured in basal conditions by the epithelial voltmeter and then converted to transepithelial conductance (TEEC) using the formula TEEC  =  1/TEER [30].

### 2.5. Scanning Electron Microscopy

Primary cultured epithelial cells were seeded in 6.5 mm Transwell^®^ with 0.4 µm-pore polyester membrane sterile insert (2 × 10^5^ cells/insert/100 µL) while 350 µL of bronchial epithelial cell growth medium (Lonza, Basel, Switzerland) was added to the basolateral side of the transwell system (12 inserts in a 24 well plate). The apical medium was removed 3 days after seeding the cells on top of the inserts. The newly established air–liquid interface (ALI) cell culture system was cultured for an additional 8–10 days to achieve full confluency. Afterward, epithelia were washed apically with 100 µL of phosphate-buffered saline (PBS). A total of 100 µL of PBS supplemented or not with each Esc peptide at 20 µM was added on the apical compartment. Cells were then incubated for 5 h at 37 °C. Afterward, apical supernatant was removed, and samples were fixed with 2.5% glutaraldehyde in 0.01 M PBS for 60 min. The samples were then fixed in 1% osmium tetroxide (OsO_4)_ for 60 min and extensively washed with the same buffer and dehydrated with a graded ethanol series. After dehydration in hexamethyldisilazane and sputter coating, the samples were examined using a scanning electron microscope (Philips XL 30 CP instrument).

### 2.6. Gene Expression

All animal experiments were carried out based on a protocol (n. 20087639) approved by the Institutional Animal Care and Use Committee of the University of Pittsburgh according to the National Institutes of Health (NIH) guide for the care and use of laboratory animals. Seven-week-old female wild-type CD1 mice were anesthetized by isoflurane inhalation and instilled intratracheally with 50 µL of peptide solution (0.1 mg/kg, i.e., 20 μM) or PVA-PLGA NP suspension (either bare NPs or NPs loaded with each peptide at 0.1 mg/kg), in PBS. Control mice were given 50 µL of PBS without peptide. After 24 h, mice were euthanized for lung tissue isolation to extract RNA for differential expression analysis (sequencing work was performed by Novogene US Marketing). 

### 2.7. Mouse Toxicity and Maximum Tolerated Dosage (MTD)

Five-week-old female wild-type CD1 mice were anesthetized by isoflurane inhalation and instilled intratracheally with 1.5 mg/kg (corresponding to 300 μM) of Esc(1-21)-1c in the free or encapsulated form in 50 μL of PBS. Mice instilled with 50 μL of PBS or bare PVA-PLGA NPs were included for control. After 1 day and 14 days, mice were euthanized for tissue isolation and examined for histopathology. Tissues were fixed in situ with 4% paraformaldehyde for 10 min with open chest cavity. They were then embedded in paraffin, and 5 µm-thick tissue slices were prepared by staining in hematoxylin and eosin and analyzed for pathological severity. In parallel, another group of seven animals was anesthetized by isoflurane inhalation and instilled intratracheally with escalating concentrations up to 7 mg/kg Esc(1-21)-1c in 50 μL of PBS and monitored for survival to determine the MTDs. 

### 2.8. Stability Measurements in Bronchoalveolar Lavage Fluid

Bronchoalveolar lavage fluid (BAL) was collected from male and female C57BL/6 mice (4–5 months old) according to the procedure described in [31,32]. Mice were euthanized by means of CO_2_ narcosis; a small incision at the level of the neck was made and a blunt needle connected to a syringe was inserted into the trachea. Then, lungs were washed with 1 mL of sterile PBS. BAL samples from each mouse were pooled, centrifuged at 1000 rpm (corresponding to 0.1× *g*) for 5 min, and stored at −80 °C. The supernatant was used for stability tests. 

Peptides were dissolved in 400 µL of PBS at a concentration of 300 µM, and added to an equal volume of BAL at a final concentration of 150 µM. At different time points, 150 μL aliquots were withdrawn and added to 500 µL of acetonitrile, and then centrifuged. The supernatants were diluted with 160 μL of 0.1% trifluoroacetic acid (TFA)—water and analyzed by RP-HPLC and mass spectrometry. Liquid chromatography was performed on a Phenomenex Jupiter C_18_ analytical column (300 Å, 5 μm, 250 × 4.6 mm) in a 30 min gradient, using 0.1% TFA in water as solvent A and acetonitrile as solvent B. Mass spectrometry analysis of diluted samples was performed with a Bruker Daltonic-ultraflex-matrix-assisted laser desorption ionization tandem time-of-flight (MALDI-TOF/TOF) mass spectrometer. 

### 2.9. Statistical Analyses

Quantitative data, collected from independent experiments, were expressed as the means ± standard errors of the means (S.E.M.). Statistical analysis was performed using one-way analysis of variance (ANOVA) with PRISM software version 8.0.1 (GraphPad, San Diego, CA, USA). Differences were considered statistically significant at a *p* value of <0.05. The levels of statistical significance are indicated in the legends of the figures.

## 3. Results

### 3.1. In Vitro Effect of Esc Peptides and Esc-Peptide-Loaded PVA-PLGA NPs on F508del-CFTR-Expressing Epithelium

**Figure 1:**
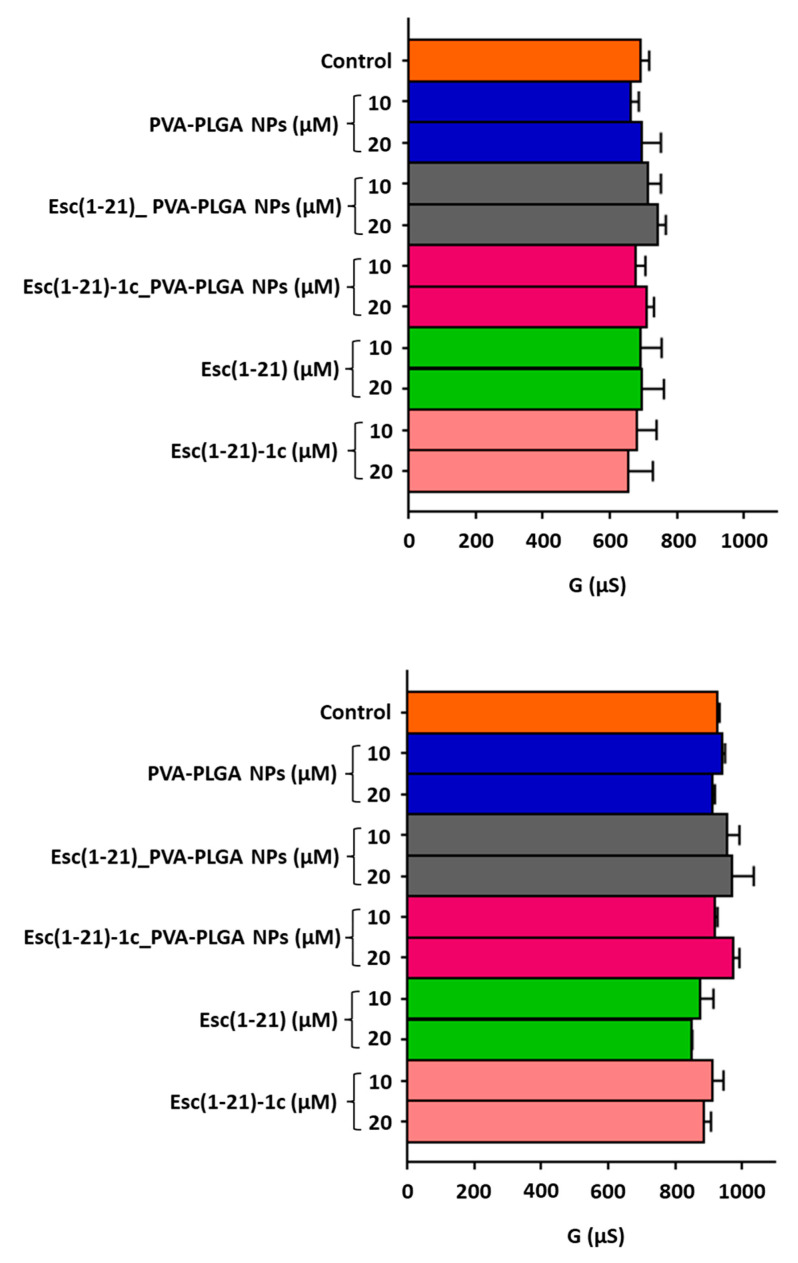
Effect of bare or Esc-peptide-loaded PVA-PLGA NPs on transepithelial conductance measured in F508del (upper panel) and wt (lower panel) CFBE41o- after 24 h of incubation compared to the free Esc peptides at two different concentrations. The controls were untreated cells. Data are expressed as mean ± S.E.M. from three independent experiments. No statistical difference was found.

Esc-peptide-loaded PVA-PLGA NPs, comprising a PLGA core to efficiently entrap and slowly release the peptide cargo and a PVA shell providing for mucus-/biofilm-penetrating properties, were produced as previously reported [27]. In the optimized formulation conditions, NPs display a hydrodynamic diameter lower than 300 nm, a low polydispersity index, a slight negative ζ-potential, a complete peptide entrapment (entrapment efficiency always around 100%), and a typical biphasic in vitro release profile of the entrapped Esc peptide, lasting for 3 days [27]. Conceiving PVA-PLGA NPs for inhalation [27], their effect on the lung epithelium integrity was initially tested, either in the free or loaded form, by measuring the transepithelial electrical conductance after 24 h of treatment. Bronchial epithelial cells expressing a functional copy of CFTR or its mutated F508del form (wt-CBFE41o- and F508del-CBFE41o-, respectively) were employed. Peptides in the soluble free form were also included for comparison. They were used at two different concentrations, i.e., 10 and 20 μM. Note that 10 μM corresponds to the best (minimal) concentration able to display a CFTR potentiator activity in bronchial epithelial cells [25], while 20 μM was the optimal peptide concentration showing pulmonary antimicrobial efficacy [26]. As reported in Figure 1, negligible changes in the transepithelial electrical conductance were attained when the epithelium was treated with the Esc-peptide-loaded PVA-PLGA NPs in comparison to untreated samples or samples treated with the free-peptide counterparts. This indicates that neither PVA-PLGA NPs or Esc peptides are harmful to epithelial cells expressing either wild-type or mutated CFTR nor cause paracellular leakage of ions, meaning that cell junctions remain well-tightened.

Remarkably, similar results were obtained for the FRT expressing F508del-CFTR, which have been extensively used for studies on CFTR protein (Figure S2). 

Note that epithelial cells may be able to repair the damage induced by the administration of exogenous compounds in the long term (24 h). However, the harmless effect of Esc peptides on both normal and CF lung epithelia was also confirmed at a shorter time (5 h) by scanning electron microscopy (SEM). As shown in Figure S3, treatment of both normal and CF primary bronchial epithelial cells (grown in ALI to better mimic the human airway conditions and to drive differentiation towards a mucociliary phenotype) with 20 μM Esc peptides did not provoke any significant morphological change in the epithelial surface.

### 3.2. In Vivo Studies: Effect of Free or Encapsulated Esc Peptides on Global Pulmonary Genetic Changes

To go in-depth into the potential clinical application of inhalable PVA-PLGA NPs as delivery systems of Esc peptides, we determined their pulmonary safety profile in healthy mice, when used either in their bare form or loaded with the Esc peptides. For a complete overview of the global genetic changes in the lungs of animals after 24 h exposure to peptide-loaded PVA-PLGA NPs compared to the free peptides or the vehicle PBS, we performed transcriptome studies by RNA-seq. To this aim, the standard immunocompetent outbred mouse strain CD-1 was used. Due to its ability to reflect the natural response of a non-immunocompromised human being, this is a common animal model for toxicity analysis. Esc-peptide-loaded PVA-PLGA NPs as well as free Esc peptides at a dosage of 0.1 mg/kg (~20 μM), which have already found to be active in vivo by provoking a 2-log_10_ reduction in lung bacterial burden [26], were intratracheally administered. 

Only a minimal number of lung genes (up to 6 out of 25,000) were up- or downregulated by more than two-fold in animals treated with Esc peptides or with Esc-peptide-loaded PVA-PLGA NPs versus PBS-treated animals. The identified genes are shown in Table 1; the corresponding volcano plots of the differential gene expression are reported in Figure S4. Among them were a member of the non-protein-coding small nucleolar RNA gene family (*Snhg11)*, three pseudogenes (i.e., *Eif4a-ps4, Gm10320*, and *Gm14150*), and two genes predicted to be structural constituents of ribosomes (i.e., *Rpl26* and *Rpl30*) or encoding a component of the cytoskeletal motor protein dynein (*Dynlt1f*) or alpha-synuclein (*Snca*), while the other genes were found to be involved in the positive regulation of protein phosphorylation (*Chil1*) or the signaling receptor-binding activity (*H2-T22*). Remarkably, none of these genes appear to be involved in toxicity-related processes.

### 3.3. Esc(1-21)-1c Safety Profile in Lungs and Other Organs

**Figure 2:**
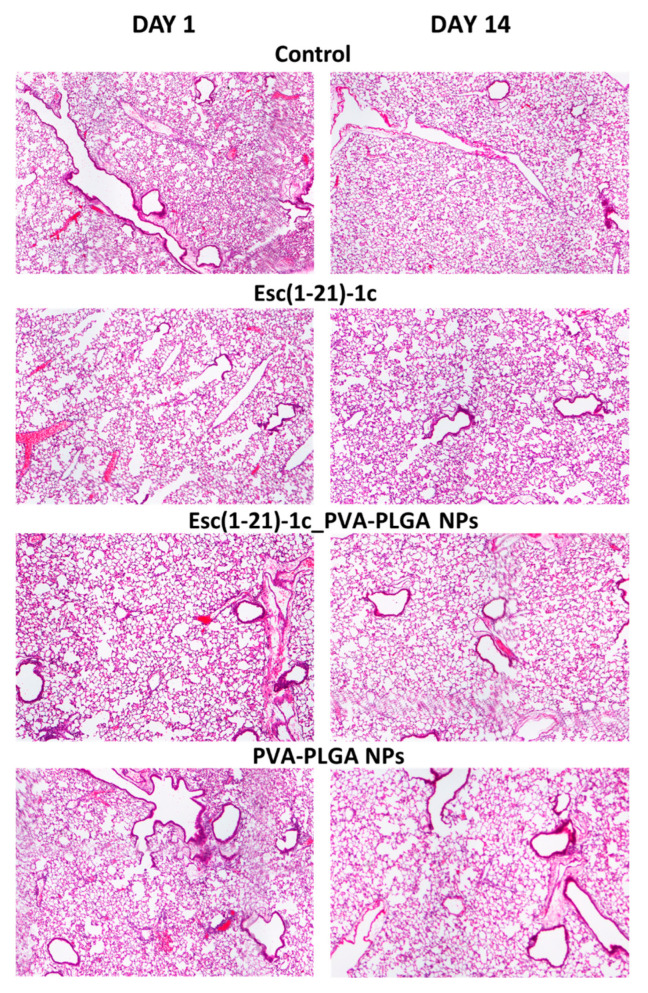
Histological analysis of lungs in CD1 mice, after 1 day and 14 days from i.t. administration of Esc(1-21)-1c either in the free or encapsulated form at a dosage of 1.5 mg/kg. The amount of unloaded PVA-PLGA NPs was the same as that present in Esc(1-21)-1c-loaded NPs. The results were compared to those obtained with PBS-treated mice (control), (magnification 4×).

We previously demonstrated that i.t. instillation of 0.1 mg/kg of Esc peptides either in the free form or upon encapsulation into PVA-PLGA NPs did not alter the lung mucociliary clearance in healthy mice [27]. However, the in vivo toxicity of Esc-peptide-loaded PVA-PLGA NPs upon pulmonary administration is an unexplored area. Therefore, we investigated the lung safety of the most promising Esc peptide, Esc(1-21)-1c, for therapeutic development. To this purpose, Esc(1-21)-1c was i.t. instilled in CD1 mice either in the free or encapsulated form at 1.5 mg/kg (~300 µM), corresponding to a 15-fold-higher concentration than that used in previous in vivo efficacy studies (0.1 mg/kg) [27]. As highlighted by the histological analysis in Figure 2, we did not detect any inflammatory response (there were no infiltrates of inflammatory cells) or lung tissue damage either after 1 day or 14 days from peptide administration in the free or encapsulated form. In parallel, no toxicity was detected for the corresponding amount of bare PVA-PLGA NPs, in line with our previous work showing an invariant expression of inflammation-associated genes (including IL-6, IL-10, or the tumor necrosis factor-α and NF-κB) in the lungs of mice after instillation of PVA-PLGA NPs encapsulated or not with Esc peptides [27].

**Figure 3:**
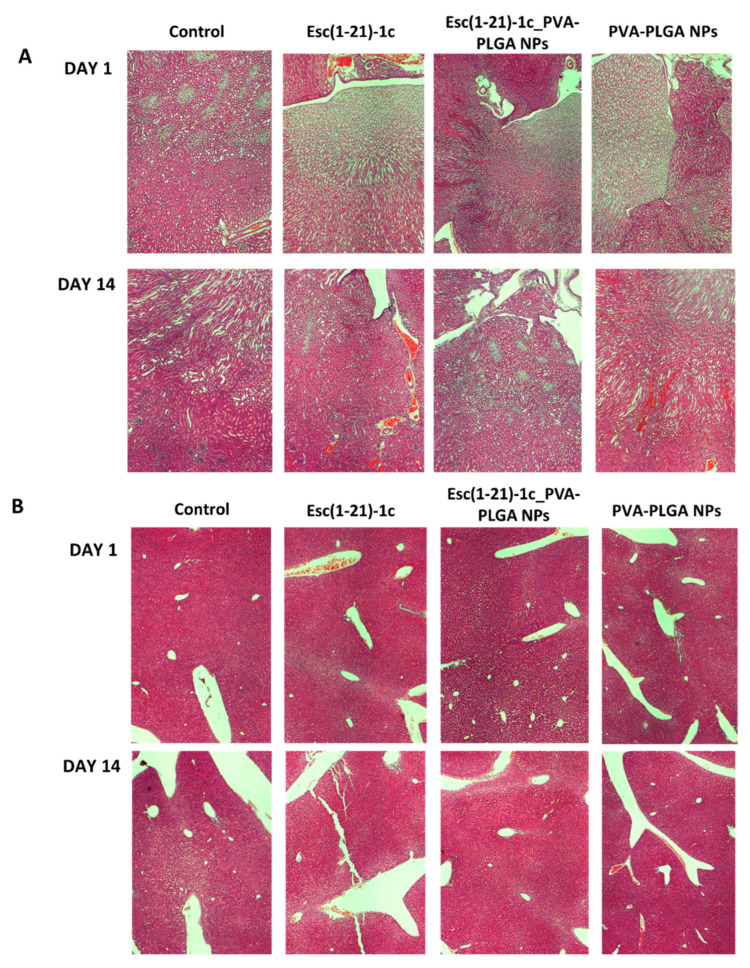
Representative images of kidney (**A**) and liver (**B**) tissues in CD1 mice, after 1 day and 14 days from i.t. administration of Esc(1-21)-1c either in the free or encapsulated form at a dosage of 1.5 mg/kg compared to the corresponding bare PVA-PLGA NPs or PBS-treated animals (control). Organs were harvested, fixed, and stained for histological evaluation (magnification 4×).

In addition, no visible tissue injury, necrosis, or alteration in cell density was observed for other organs, such as the liver and kidneys, in comparison to control samples (Figure 3). 

**Figure 4:**
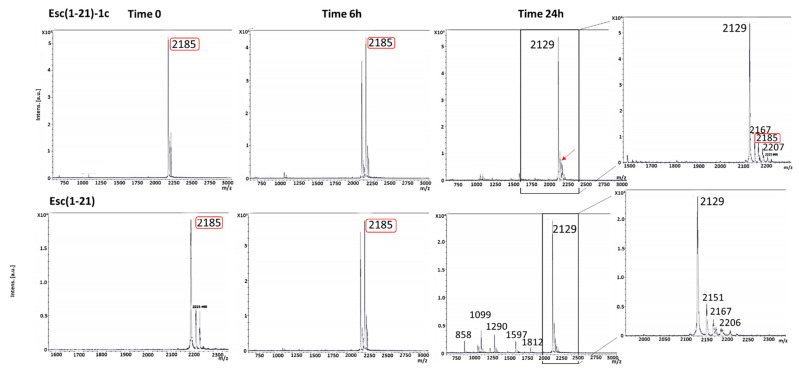
Mass spectra of Esc(1-21)-1c and the all-L Esc(1-21) after 6 and 24 h in BAL. Marked in red is the peak of molecular mass at 2185 corresponding to the full-length peptide. The arrow indicates the peak of molecular mass at 2185 found for Esc(1-21)-1c after 24 h of incubation with BAL.

To ensure that the lung safety of Esc(1-21)-1c 24 h after its administration was not due to its complete degradation in the lung environment, we studied its biostability at 300 μM in the presence of mouse BAL. As pointed out by the mass spectra in Figure 4, when Esc(1-21)-1c was incubated with BAL, a peak of molecular mass of 2185 Da corresponding to the full-length peptide [22] was detected even after 24 h. On the contrary, the all-L isomer was significantly less stable, as highlighted by the appearance of multiple degradation products and the lack of the peak corresponding to the entire peptide sequence after 6 h. However, both spectra showed the appearance of a peak (2129 Da) at 6 h, which became prevalent at 24 h. This peak indicates the loss of a Gly residue from the peptide sequences.

### 3.4. Determination of Maximum Tolerated Dosage 

Development of peptides as anti-infective agents requires knowledge of their therapeutic index (TI), which is defined as the ratio between the maximum tolerated dosage, MTD (i.e., the highest concentration causing no obvious adverse effects and no mortality), and the therapeutic dosage mTd (i.e., the minimal dosage reducing bacterial burden by 2-log_10_ in the number of bacterial cells) [33]. Therefore, to identify the MTD of the most efficacious Esc(1-21)-1c, CD1 mice were i.t. instilled with increasing concentrations of the peptide, from 1.5 mg/kg to 7 mg/kg, and their survival was monitored for 14 days. Remarkably, the peptide was well-tolerated by the animals, which remained viable for the entire duration of the experiment and maintained the same range of motion both after a short time (1 h) and longer time (24 h) from its i.t. instillation at the highest dosage of 7 mg/kg. Notably, this concentration was 70-fold greater than the efficacious dose, indicating a TI of at least 70. When such a high concentration of Esc(1-21)-1c was used, the peptide was detectable in the mouse BAL after 48 h of incubation with BAL (Figure S5). This finding indicates that the harmlessness of the peptide at the long term cannot be attributed to its degradation.

**Figure 5:**
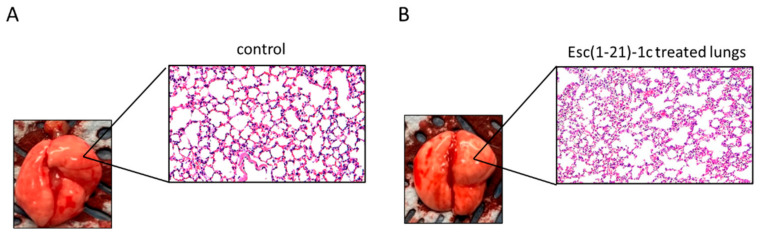
Histologic analysis of mouse lung tissues at 48 h after i.t. instillation of Esc(1-21)-1c at 7mg/kg. Lung tissues were harvested, fixed, and stained for histological evaluation without peptide treatment (**A**) or after peptide administration (**B**) (magnification 20×).

Furthermore, histopathological examination of lung tissue did not reveal significant recruitment of inflammatory cells compared to PBS-treated mice within 48 h from i.t. instillation of Esc(1-21)-1c, in agreement with the absence of macroscopic damage to the lungs (Figure 5). 

**Figure 6:**
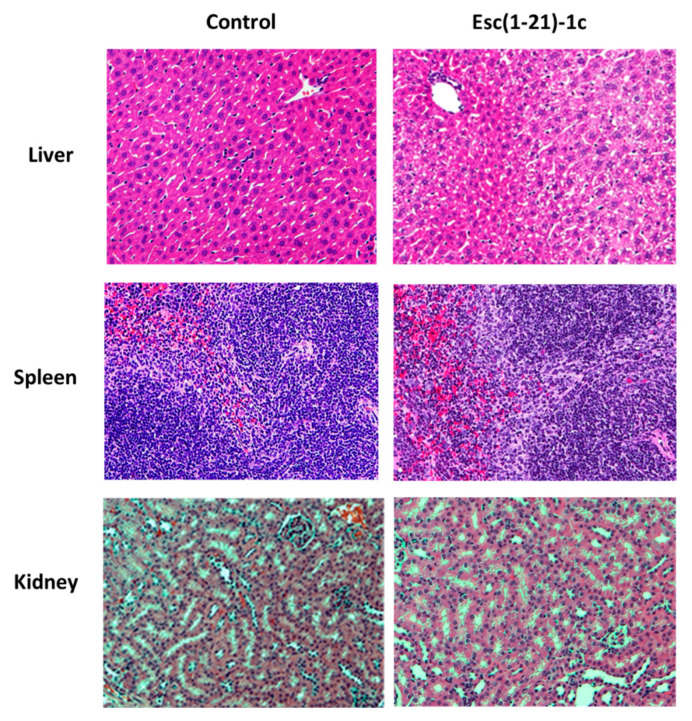
Representative images of liver, spleen, and kidneys from healthy mouse at 48 h after i.t. instillation of Esc(1-21)-1c at 7mg/kg. Tissues were harvested, fixed, and stained for histological evaluation without peptide treatment (control, left side) or after peptide administration (right side) (magnification 20×).

Similarly to what was found for the peptide dosage of 1.5 mg/kg, no alteration in the tissue structure was visible at the level of the liver, spleen, and kidneys after treatment with 7 mg/kg of Esc(1-21)-1c, compared to the control (Figure 6).

## 4. Discussion

The urgent health concern related to antibiotic resistance has driven a renewed interest in the clinical development of peptides compared to the previous two decades. Interestingly, besides having direct antimicrobial activity, some AMPs also display additional biological functions involved in the modulation of host immunity, including enhanced chemotaxis of immune cells, activation of immune cell differentiation, stimulation of wound healing, and angiogenesis, scavenging of bacterial endotoxins [34]. The broad spectrum and rapid bactericidal activity of AMPs combined with the low risk of inducing resistance makes them a valid option for alternative antimicrobial compounds. However, to the best of our knowledge, there are no AMPs in clinical trials for the development of new inhalable drugs against lung infections.

Here, to emphasize the attractive properties of Esc peptides for the treatment of lung diseases, especially in CF, likely via pulmonary delivery upon incorporation into PVA-PLGA NPs, we investigated their effect on the lung epithelial integrity and the host immune response in terms of gene expression and tissue damage at the target district. Remarkably, we discovered that both Esc peptides and PVA-PLGA (drug carrier) are harmless to epithelial cells. This was evidenced by the invariant transepithelial conductance in bronchial epithelia upon treatment, as measured by TEER experiments, which appear to be more sensitive than typical cytotoxicity tests by the 3-(4,5-dimethylthiazol-2-yl)-2,5-diphenyltetrazolium bromide (MTT) assay. Nonetheless, one of the most critical limitations of AMPs, particularly when embedded into a delivery system, is the lack of potential toxicity data in animal models. There are currently 15 FDA-approved PLA-/PLGA-based drug products available on the US market [35], and several studies on the usage of PLGA-based nanocarriers for pulmonary drug delivery are in progress [36,37]. Together with chitosan, PLGA is the most investigated polymer for the development of inhaled formulations aimed at extending the pulmonary exposure and pharmacological effect of encapsulated drugs [38]. This is because of its biodegradability [39], which can be modulated by varying the lactide/glycolide ratio, molecular weight, chemical structure, and biocompatibility [28]. We formerly highlighted how incorporating AMPs into PVA-PLGA NPs and their pulmonary administration represent a suitable approach to assist peptide diffusion through an artificial lung mucus, eradicate biofilm, and potentiate and extend the in vivo antimicrobial efficacy of Esc peptides in the lung [27]. The work from Haque and colleagues [40] reported that PLGA NPs do not appear to be absorbed into the lungs after pulmonary administration but rather are degraded into lower-molecular-weight constituents that are subsequently absorbed. PLGA NPs have also been used (i) to release loaded AMPs to the wounds to accelerate healing processes [41] and (ii) to deliver drugs in tumor combination therapy [42].

Nevertheless, a limited number of studies have been performed to confirm the safety of PLGA NPs in the short and long term, especially in the lung compartment [43,44,45,46]. 

Our previous work highlighted that PVA-PLGA NPs do not induce any recruitment of inflammatory cells into the lung alveoli of healthy mice nor changes in the expression of inflammation-associated genes 36 h after pulmonary administration [27]. Here, the transcriptomic analysis provided us with the first demonstration that Esc peptides, either in the free or encapsulated form in PVA-PLGA NPs, promote a response closer to the gene expression pattern of the PBS-treated host. 

To the best of our knowledge, this is the first case showing the effect of peptide-loaded PVA-PLGA NPs on the global genetic profile of targeted tissues (i.e., lungs) upon administration in the conductive airways of mice, in comparison to the vehicle-treated animals. Interestingly, only 2 genes out of 25,000 were found to be up-/downregulated by more than 2-fold by the Esc-peptide-loaded PVA-PLGA versus the PBS-treated mice (i.e., *H2-T22* and the pseudogene *Gm10320* for Esc(1-21)-loaded NPs and *Snca* and the pseudogene *Gm14150* for Esc(1-21)-1c-loaded NPs). Moreover, as pointed out by histological analysis of lungs tissue, no sign of inflammation/damage was detected by NPs when used in the free or loaded form with Esc(1-21)-1c at a concentration 15-fold higher than the therapeutic dosage of 0.1 mg/kg [47,48,49], either after 1 day or 14 days from i.t. administration. Together, these data have contributed to further supporting the harmlessness of PVA-PLGA NPs in the respiratory tract.

The other important finding of this work is the discovery of the safety profile of Esc(1-21)-1c and its tolerability by animals, at a concentration 70-fold higher than the efficacy dose, without eliciting any detectable damage to the lung, spleen, liver, and kidney, unlike what has been described for the AMP D-BMAP18, which was found to cause lung edema when used at doses of 1 and 2 mg/kg [50]. In addition, we proved that the absence of toxicity was not due to the complete degradation of the peptide, which instead remained available in the BAL within the first 48 h. This is in contrast with the low biostability of other investigated AMPs for treatment of lung infections, such as (i) all-L-BMAP18 AMPs, which were degraded by pulmonary proteases in murine BAL fluids within the first 20 min of exposure [51] and did not display any in vivo antibacterial activity, and (ii) P-113, which showed a half-life of a few minutes in undiluted sputum [52]. 

## 5. Conclusions

In conclusion, we have filled the gap of some underexplored but relevant aspects for the translation of AMPs into new inhalable medicines by providing evidence of (i) the pulmonary safety profile of Esc peptides and PVA-PLGA NPs as a valuable nanoparticulate system for AMP delivery to the lungs; (ii) a prolonged biostability of Esc(1-21)-1c in the mouse BAL together, and (iii) its promising therapeutic index.

Overall, in addition to expanding our knowledge on the safety profile of Esc peptides for the development of new drugs to treat *P. aeruginosa* lung infection, our studies should open the avenue for a new up-and-coming pharmacological approach, likely based on inhalable peptide-loaded NPs, to address CF lung disease.

## Figures and Tables

**Table 1:** List of up- or downregulated genes in lungs of animals treated with Esc(1-21)-1c, Esc-peptide-loaded PVA-PLGA NPs, compared to those of PBS-treated animals.

	Effect	Gene Name	Gene Description	Gene Type	Log_2_FC
Esc(1-21)	None				
Esc(1-21)-1c	Down	*Snhg11*	Small nucleolar RNA host gene 11	Protein coding	−1.949
	Up	*Rpl30*	Ribosomal protein L30	Protein coding	14.876
	Up	*Rpl26*	Ribosomal protein L26	Protein coding	20.049
	Up	*Chil1*	chitinase-like 1	Protein coding	34.248
	Up	*Eif4a-ps4*	-	Pseudogene	17.867
	Up	*Dynlt1f*	Dynein light-chain Tctex-type 1F	Protein coding	19.702
Esc(1-21)_PVA-PLGA NPs	Down	*H2-T22*	Histocompatibility 2 T region locus 22	Protein coding	−15.437
	Up	*Gm10320*	Predicted pseudogene 10320	Protein coding	24.607
Esc(1-21)-1c_PVA-PLGA NPs	Down	*Snca*	Synuclein alpha	Protein coding	−14.029
	Down	*Gm14150*	Predicted gene 14150	Pseudogene	−25.684

Up: upregulated; down: downregulated. Log_2_ fold change is also indicated.

## Data Availability

Data are contained within the article or Supplementary Material.

## Supplementary Materials

The following supporting information can be downloaded at: https://www.mdpi.com/article/10.3390/pharmaceutics14112297/s1. Figure S1: HPLC and mass spectra of Esc peptides; Figure S2: Effect of bare or Esc-peptide-loaded PVA-PLGA NPs on F508del-FRT epithelium integrity after 24 h of incubation; Figure S3: SEM images of bronchial epithelia; Figure S4: Effect of free or encapsulated peptides on lung gene expression; Figure S5: Mass spectra of Esc(1-21)-1c after 48 h of incubation with mouse BAL.
