# Supplementary material for: Pulmonary Safety Profile of Esc Peptides and Esc-Peptide-Loaded Poly(lactide-co-glycolide) Nanoparticles: A Promising Therapeutic Approach for Local Treatment of Lung Infectious Diseases
Source: Pharmaceutics. 2022 Oct 26;14(11):2297. doi: 10.3390/pharmaceutics14112297 (PMC9697339; doi:10.3390/pharmaceutics14112297)
Supplement: Supplementary file 1 [file pharmaceutics-14-02297-s001.zip › pharmaceutics-1947874-supplementary.pdf]

Supplementary Materials

Article

Pulmonary Safety Profile of Esc Peptides and Esc-Peptide-Loaded Poly(lactide-co-glycolide) Nanoparticles: A Promising Therapeutic Approach for Local Treatment of Lung Infectious Diseases

Floriana Cappiello [1,†], Bruno Casciaro [1,†], Maria Rosa Loffredo [1], Elena Puglisi [1], Qiao Lin [2], Dandan Yang [2], Gemma Conte [3], Ivana d'Angelo [4], Francesca Ungaro [3], Loretta Ferrera [5], Raffaella Barbieri [6], Laura Cresti [7], Alessandro Pini [7], Yuanpu Peter Di [2,*] and Maria Luisa Mangoni [1,*]

1 Department of Biochemical Sciences, Laboratory Affiliated to Istituto Pasteur Italia-Fondazione Cenci Bolognetti, Sapienza University of Rome, 00185 Rome, Italy
2 Department of Environmental and Occupational Health, University of Pittsburgh, Pittsburgh, PA 15261, USA
3 Department of Pharmacy, University of Napoli Federico II, 80131 Napoli, Italy
4 Department of Environmental, Biological and Pharmaceutical Sciences and Technologies (DiSTABiF), University of Campania Luigi Vanvitelli, 81100 Caserta, Italy
5 U.O.C. Genetica Medica, IRCCS, Istituto Giannina Gaslini, 16147 Genoa, Italy
6 Biophysic Institute, Consiglio Nazionale delle Ricerche (CNR), 16149 Genoa, Italy
7 Department of Medical Biotechnologies, University of Siena, 53100 Siena, Italy
* Correspondence: peterdi@pitt.edu (Y.P.D.); marialuisa.mangoni@uniroma1.it (M.L.M.)
† These authors contributed equally to the work.

Esc(1-21)

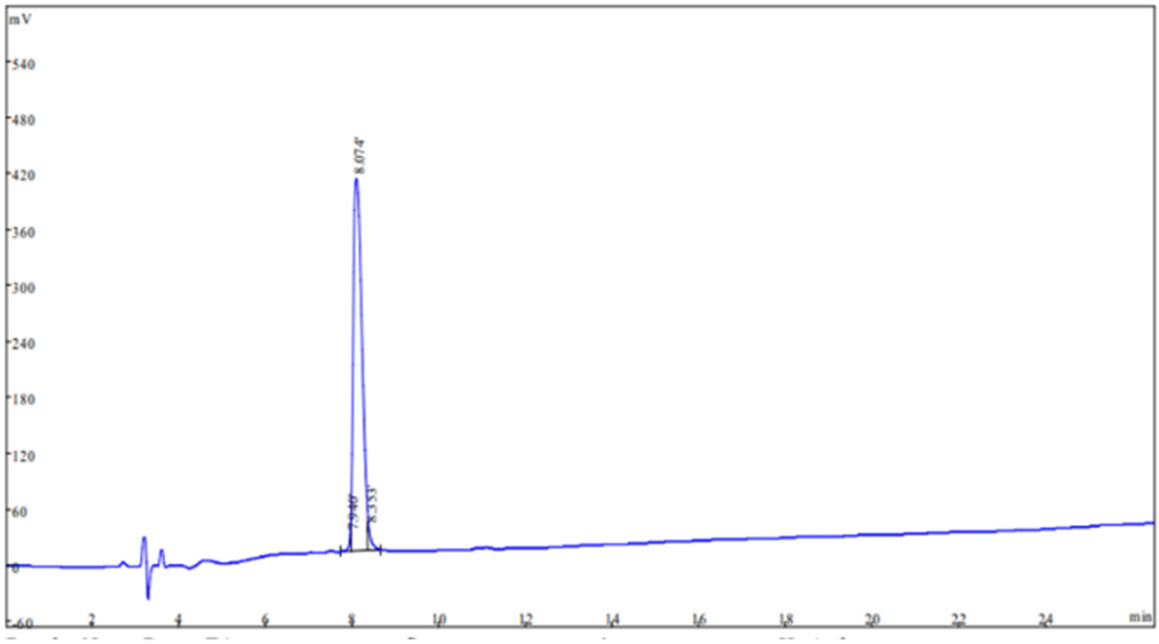

Esc(1-21)-1c

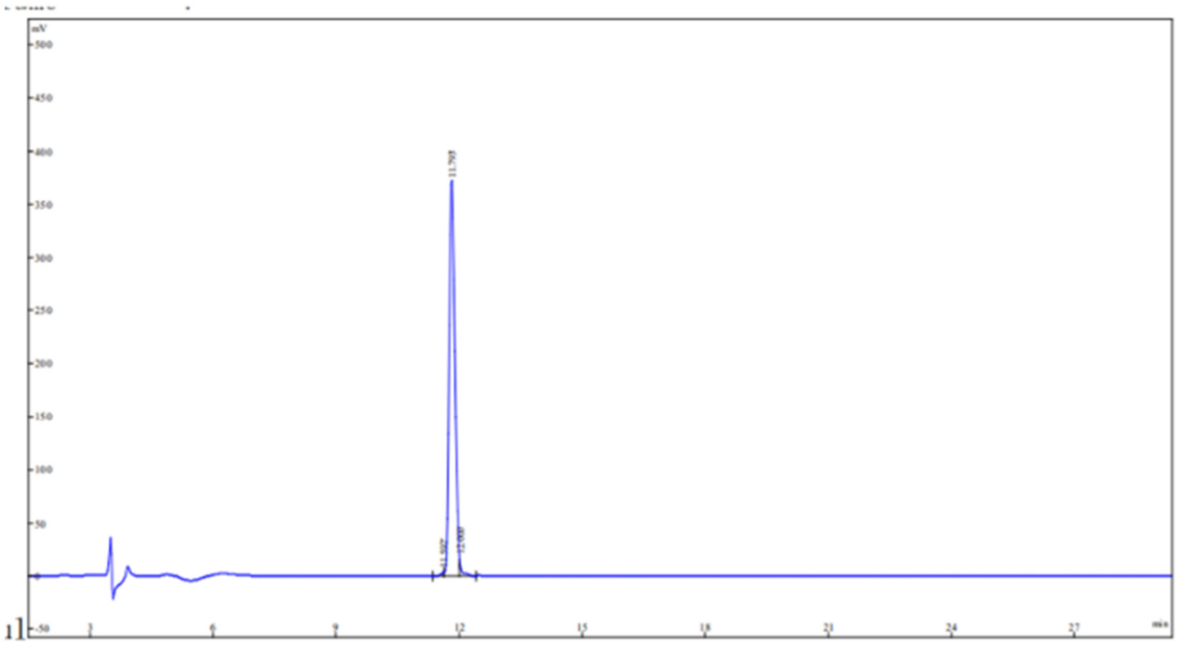

Esc(1-21)

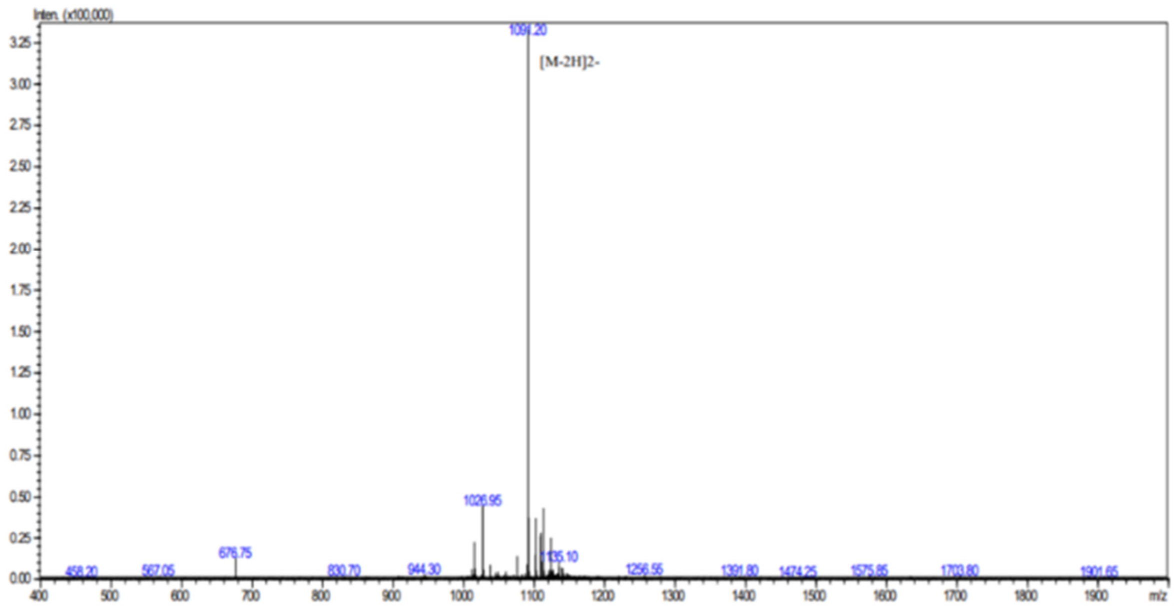

Esc(1-21)-1c

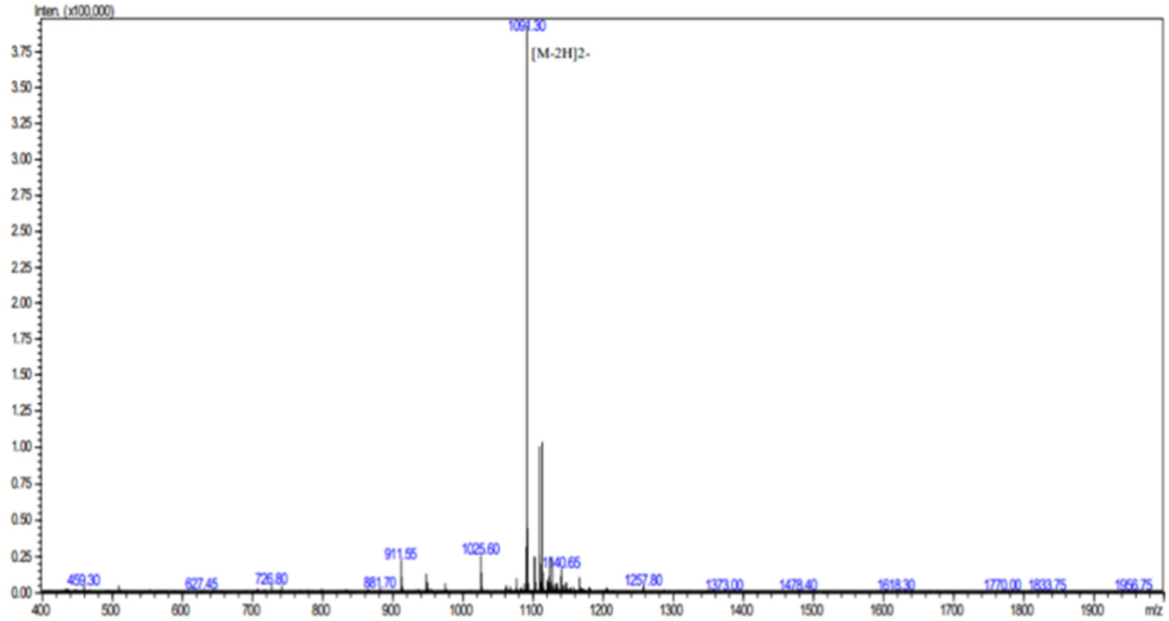

Figure S1. HPLC and mass spectra of Esc peptides.

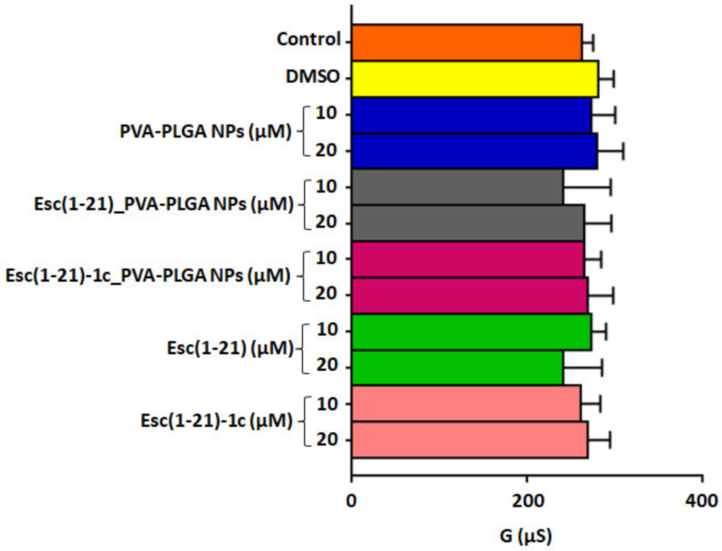

Figure S2. Effect of bare or Esc peptide-loaded PVA-PLGA NPs on F508del-FRT epithelium integrity after 24 h incubation. Untreated cells (control) and peptides in the soluble free form were also reported for comparison. Data are expressed as mean ± S.E.M. from three independent experiments. No statistical difference was found.

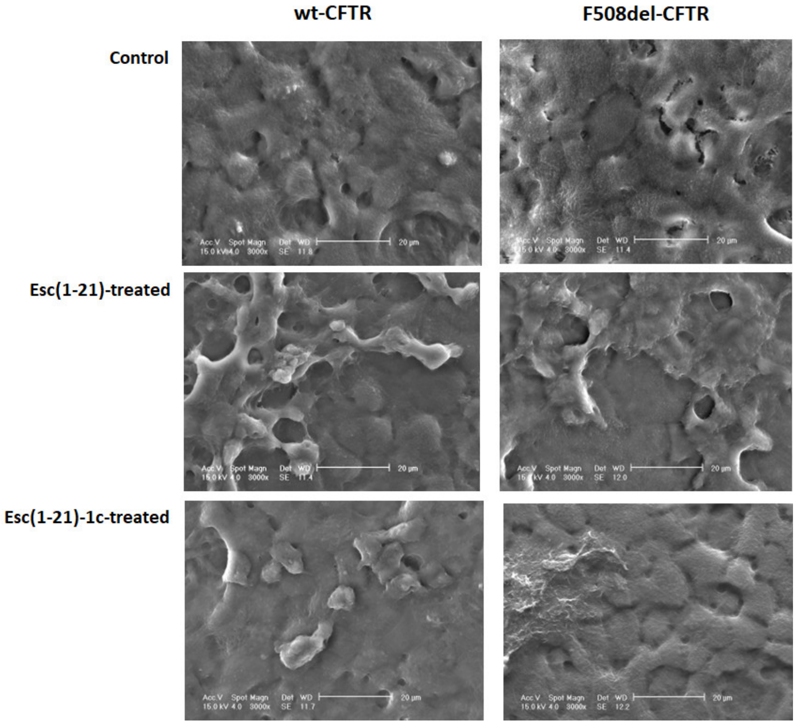

Figure S3. SEM images of bronchial epithelia. Wild type (wt) and F508del-bronchial epithelium were treated for 5 h with 20 μM Esc peptides and compared to untreated samples. Scale bar, 20 μm.

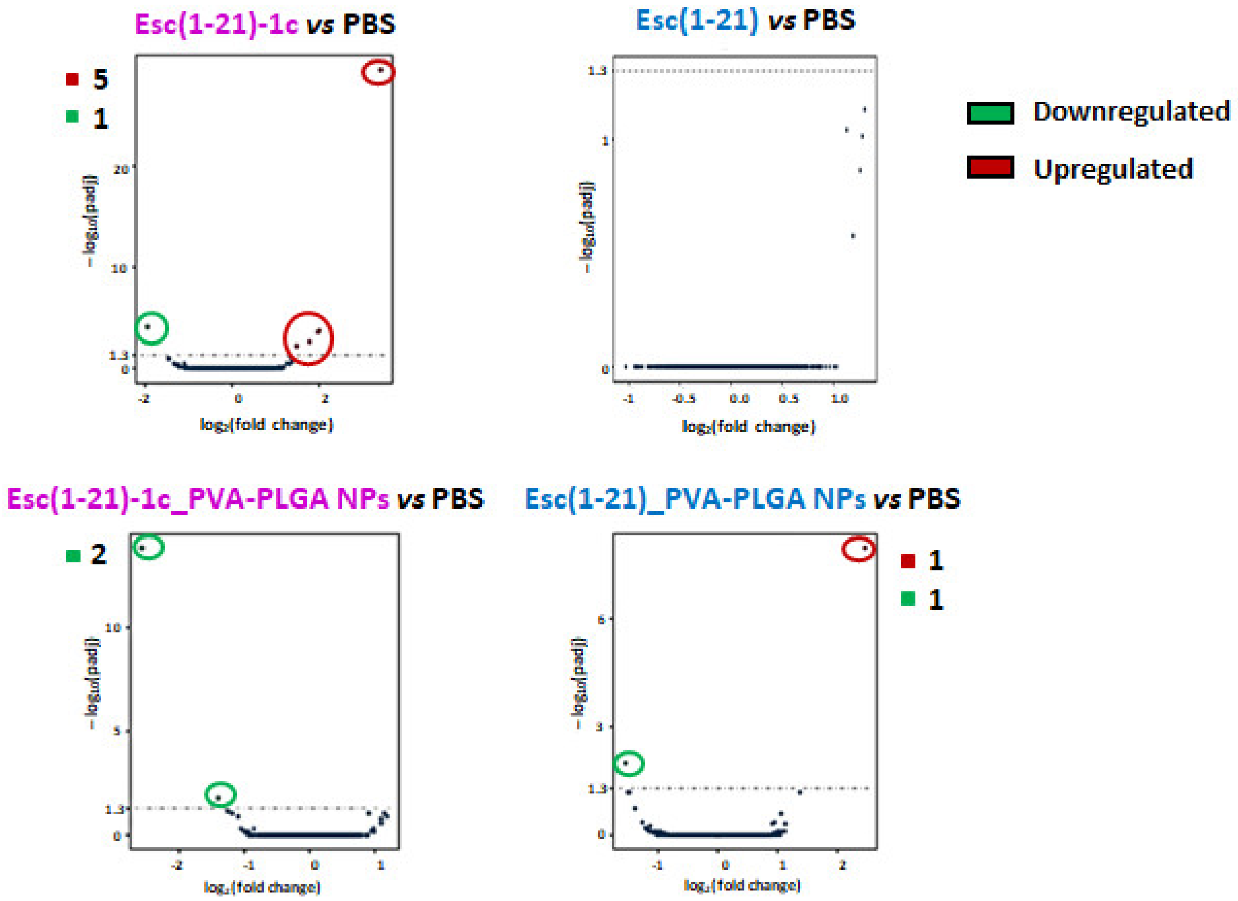

Figure S4. Effect of free or encapsulated peptides on lung gene expression. Volcano plots show differentially expressed genes at a threshold over 2-fold changes with p value < 0.05 from three biological replicates.

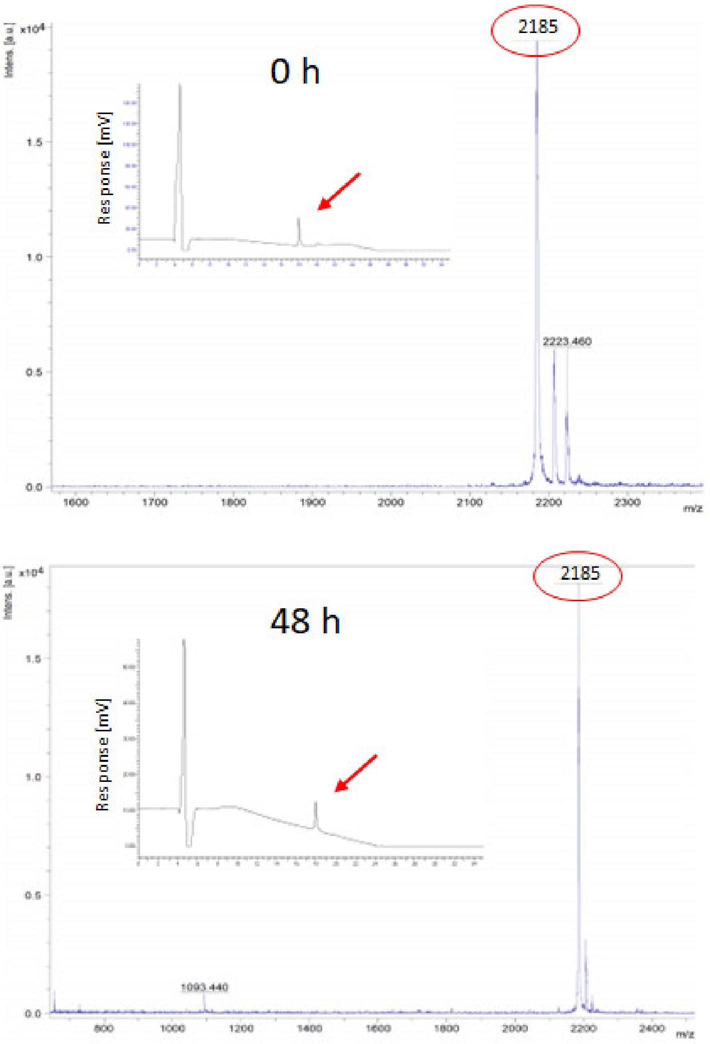

Figure S5. Mass spectra of Esc(1-21)-1c after 48 h incubation with mouse BAL. The peak of molecular mass of 2185 corresponding to the full-length peptide is marked in red. The arrows indicate the peak of 2185 molecular mass found for Esc(1-21)-1c at time 0 and after 48 h incubation with BAL.
